# ‘Accelerated’ Deactivation of Carbon Nitride Photocatalyst for Solar Hydrogen Evolution

**DOI:** 10.1002/cssc.202400937

**Published:** 2024-08-07

**Authors:** Mu Xiao, Miaoqiang Lyu, Zitong Wang, Lianzhou Wang

**Affiliations:** ^1^ School of Chemical Engineering Australian Institute for Bioengineering and Nanotechnology The University of Queensland Brisbane Queensland 4072 Australia

**Keywords:** accelerated test, carbon nitride, deactivation, stability, photocatalyst

## Abstract

Carbon nitride photocatalysts are among the most studied candidates for efficient solar hydrogen (H_2_) production due to their abundance of precursors, suitable bandgap, and visible light utilization. However, the polymeric nature of carbon nitride materials raises concerns regarding the self‐decomposition during photocatalytic redox processes. Yet, the operational stability of carbon nitride photocatalysts for solar H_2_ production remains under‐explored. Here we evaluate the photostability of carbon nitride photocatalysts with platinum (Pt) as the co‐catalyst for solar H_2_ evolution and significant deactivation of this photocatalyst is observed under'accelerated’ testing conditions. It is demonstrated that the detachment of the Pt co‐catalyst on the surface of carbon nitride is the major reason for this deactivation, which can be attributed to a synergistic effect of photo‐corrosion and mechanical stirring. The photo‐corrosion weakens the interfacial bonding between carbon nitride and Pt co‐catalyst, while continuous collisions from the mechanical stirring promote the detachment of co‐catalysts from the surface of carbon nitride. These understandings provide insights into the rational design of photocatalysts and photocatalytic systems for improved operational stability.

## Graphical abstract

An ‘accelerated test’ using high‐intensity light source is demonstrated as an effective strategy to evaluate the photostability of platinized carbon nitride photocatalysts. The photo‐corrosion combined with the collision from mechanical stirring, lead to the detachment of platinum (Pt) co‐catalysts from the surface of carbon nitride frameworks, resulting in significant deactivation for photocatalytic H_2_ evolution.

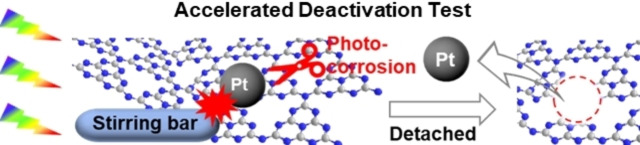

## Introduction

Solar‐powered photocatalytic H_2_ production, which converts abundant solar energy to renewable and storable H_2_ fuel, provides a promising approach to addressing energy, resource, and environmental challenges facing our human community.[^1^, ^2^] The family of semiconducting carbon nitride materials is one of the most promising photocatalysts for solar‐to‐H_2_ conversion, due to their advantageous features of visible‐light response, appropriate bandgap alignment, and earth abundance of constituent elements.[^4^, ^5^, ^6^] Despite remarkable advancement in improving efficiency for solar H_2_ production since the pioneering work reported in 2009,^[3]^ the photostability of carbon nitride photocatalysts is another equally important prerequisite for practical implementation.^[7]^ Due to the excellent thermal and physicochemical stabilities of carbon nitride materials, the photostability of carbon nitride photocatalysts has been usually overlooked and under‐explored.^[3]^ Recently, the self‐decomposition of carbon nitride photocatalysts has been observed for the CO_2_ reduction reaction and pollutant degradation.[^8^, ^9^, ^10^] Unlike pure carbon nitride photocatalysts used in these reactions, co‐catalysts are usually loaded on the surface of carbon nitride to facilitate the H_2_ evolution reaction. Whether the self‐decomposition of carbon nitride frameworks will occur for solar H_2_ evolution and the impact of this self‐decomposition on the co‐catalyst remains unclear.

Generally, the photostability of carbon nitride photocatalysts for H_2_ evolution was demonstrated by a relatively short‐term (<100 h) cycling test under mild conditions (*e. g*. visible light λ>420 nm).^[3]^ Actually, a lifetime of 5~10 years is expected for photocatalysts to achieve cost‐effective H_2_ production.[^13^, ^14^] However, a long‐term stability test (>1000 h) under operational conditions makes it less time‐efficient to identify possible deactivation, thus retarding the improvement of photostability.[^11^, ^12^] Some initial works have demonstrated high‐intensity light as an alternative and effective strategy to accelerate the long‐term stability tests for photoelectronic devices and photocatalysts, namely accelerated test.^[7]^ For instance, a 50 % loss of initial activity was detected on the GaN : ZnO photocatalyst for overall water splitting after a 6‐month operation under visible light irradiation (λ>400 nm), while a similar deactivation activity was detected within 60 hours using a high‐intensity Hg lamp (450 W, λ>300 nm).[^11^, ^12^] Thus, it is possible to use this strategy to assess the operational stability of carbon nitride photocatalysts for solar H_2_ generation.

Herein, we observe significant deactivation of three types of carbon‐nitride‐based photocatalysts with Pt as the co‐catalyst for H_2_ evolution by evaluating the photostability of these photocatalysts under the illumination of high‐intensity light (~610 mW/cm^2^, λ>300 nm), namely the ‘accelerated’ testing condition. Detailed characterizations demonstrate that the loss of the Pt co‐catalyst is the direct cause of the photocatalyst deactivation. Subsequently, we explore the influencing parameters of the reaction conditions on the stability of carbon nitride photocatalysts, including temperature, wavelength and intensity of light, type of electron donors, and mechanical stirring. The detachment of Pt co‐catalysts from the surface of carbon nitride can be attributed to a synergistic effect of photo‐corrosion and mechanical stirring. These findings provide vital insight into the future design of photocatalysts and the reaction systems towards more durable solar energy conversion. This work also demonstrates the ‘accelerated test’ as an effective strategy to assess the operational stability of photocatalysts for solar energy conversion.

## Results and Discussion

Three typical types of carbon nitride samples were synthesized to investigate the stability of carbon nitride photocatalysts for H_2_ evolution. Ionic carbon nitride (ICN) material was prepared using a modified ionothermal synthetic method, and samples via direct thermal polymerization of melamine and urea were named MCN and UCN, respectively.[^5^, ^6^, ^15^] The crystal structures and optical properties of ICN, MCN and UCN were characterized by X‐ray diffraction (XRD), Fourier‐transform infrared spectroscopy (FTIR) and ultraviolet‐visible (UV‐vis) absorption spectra, respectively (Figure S1–S3). The intense peak at 8.1° of the XRD pattern manifests the formation of ICN with a heptazine‐imide unit, while the peaks at ~13° and ~27° indicate the formation of graphite carbon nitride with a tri‐s‐triazine unit.^[15]^ In addition, UV‐vis absorption spectra confirm the visible‐light‐responsive properties of ICN, MCN, and UCN.

**Figure 1:**
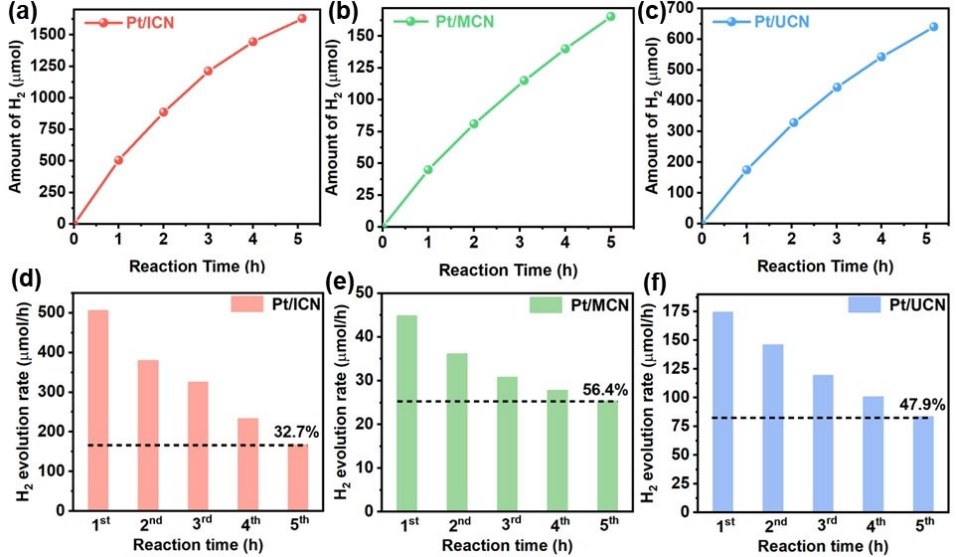
Photocatalytic H_2_ evolution activity of (a, d) ICN, (b, e) MCN, and (c, f) UCN, respectively. Reaction condition: Xe lamp (~610 mW/cm^2^, λ>300 nm), 20 mg of catalyst, 50 mL of 10 vol . % TEOA aqueous solution. 3 wt . % of Pt, magnetically stirring speed 200 rpm, temperature 20 °C, atmospheric pressure.

To validate the testing equipment and method for our photocatalytic H_2_ evolution experiment, commercially available TiO_2_ (anatase) nanoparticles with high photocatalytic stability were adopted as a reference.^[16]^ A linear increase of the H_2_ evolution amount over 5 hours verifies the accuracy of the experimental setup and measurement (Figure S4). The typical testing protocol of the photocatalytic H_2_ evolution measurement using carbon nitride materials has been adopted in this work to investigate the photocatalytic stability, of which 3 wt . % of Pt was loaded on carbon nitride photocatalysts as the co‐catalyst and a 10 vol . % of triethanolamine (TEOA) dissolved in water acted as the sacrificial electron donor.[^5^, ^6^, ^17^, ^18^] Besides, the photocatalytic reaction was operated at 1 atm of pressure with the temperature kept at 20 °C, which was preferred for practical applications.^[19]^ Specifically, a high‐intensity Xenon (Xe) lamp (~610 mW/cm^2^, λ>300 nm, Figure S5) was used as the light source to perform the ‘accelerated test’.[^11^, ^12^] More details have been described in the experimental section of the supplementary information. As shown in Figure 1, 67.3 %, 43.6 %, and 52.1 % loss of initial activity were detected after a reaction period of 5 h for ICN, MCN and UCN, respectively. Due to its highest loss of the photocatalytic H_2_ evolution rate, the ICN was selected as a typical example to study the underlying deactivation mechanism.

**Figure 2:**
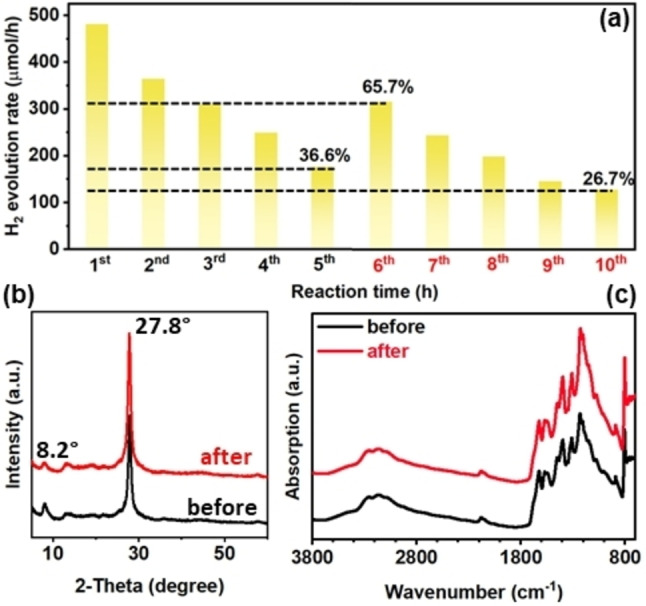
(a) Photocatalytic H_2_ evolution activity of Pt/ICN for two cycles. For the H_2_ evolution rate at the 6^th^ hour, the catalyst was collected after the 5^th^ hour and re‐dispersed in a fresh solution for the photocatalytic H_2_ evolution reaction under the same test condition. (b) XRD patterns and (c) FTIR spectra of Pt/ICN before and after the 5 h‐photocatalytic H_2_ evolution reaction.

A possible reason for the decreased H_2_ evolution rate can be the change in the local environment around the photocatalyst, such as the pH value and the accumulation of products.[^11^, ^12^] In particular, the accumulation of H_2_ inside the reactor under atmospheric pressure, may impede the desorption of H_2_ bubbles on the surface of the photocatalyst, which in turn prevents the photocatalyst from further catalysing the reactions.^[20]^ In this regard, the photocatalyst after the five‐hour reaction was collected, washed, and re‐dispersed into a fresh solution for the second‐cycle test. The H_2_ evolution rate of the re‐dispersed Pt/ICN recovered to 66.7 % of the initial value and decreased continuously with a much lower speed, reaching 26.7 % of the initial value after another five‐hour testing (Figure 2a). The pH values of the reaction solution before and after the 5‐h photocatalytic reaction were ~10 and ~10.5, respectively. These results suggest that the deactivation is partially caused by the change of the local environment around the photocatalyst, most probably the adsorption of H_2_ bubbles on the active sites of the photocatalyst.

**Figure 3:**
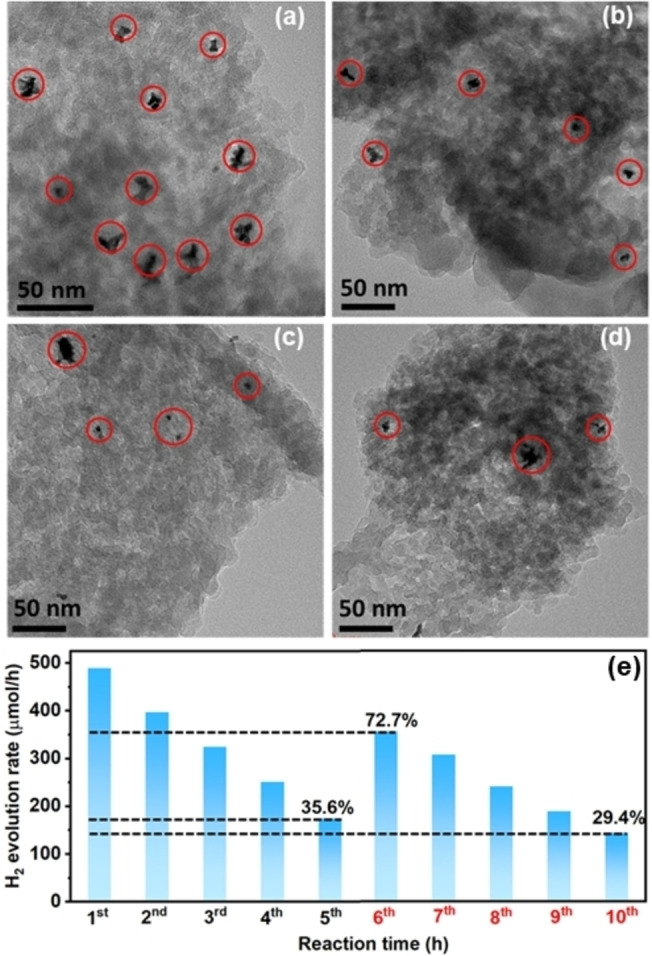
HRTEM images of Pt/ICN photocatalysts (a, b) before and (c, d) after the H_2_ evolution reaction, respectively. (e) Photocatalytic H_2_ evolution rate of Pt/ICN with the addition of extra Pt to regenerate the active sites. 6^th^: the tested catalyst is collected and re‐dispersed in a fresh solution with the addition of 1 wt . % Pt precursor.

As the photocatalytic performance of Pt/ICN is not fully recovered to the initial state after re‐dispersing in a fresh solution (Figure 2a), we assume that the possible reasons may be related to the structure and property changes of the tested photocatalyst.[^11^, ^12^] Compared to the pristine ICN, there are no obvious structural changes detected on the deactivated Pt/ICN using XRD (Figure 2b), FTIR (Figure 2c), scanning electron microscopy (SEM, Figure S6), and X‐ray photoelectron spectroscopy (XPS, Figure S7), respectively. Nevertheless, it was noticed that the colour of the magnetic stirring bar turned from white to dark after a series of photocatalytic tests. The dark coating on the stirring bar was identified to be Pt from energy‐dispersive X‐ray spectroscopy (EDS, Figure S8), which might come from the detachment of the Pt co‐catalyst on ICN. To confirm whether the Pt originates from the detachment of the carbon nitride photocatalysts, high‐resolution transmission electron microscopy (HRTEM) was performed to compare the morphology of the photocatalysts before and after photocatalytic reactions. Compared with the initial Pt/ICN, much less Pt nanoparticles are identified on the tested photocatalyst, suggesting the loss of Pt nanoparticles from the Pt/ICN photocatalyst during the reactions (Figure 3a–d). According to the quantitative analysis from inductively coupled plasma optical emission spectroscopy (ICP‐OES), the elemental ratios of Pt on ICN are identified to be 3.04 wt . % and 1.03 wt . % for initial Pt/ICN and tested Pt/ICN, respectively, which further evidences the detachment of the Pt co‐catalyst from the surface of ICN after reactions. The loss of the Pt co‐catalyst may also explain lower absorbance between 450–800 nm for deactivated Pt/ICN, compared to that of original Pt/ICN (Figure S9). As the photocatalytic H_2_ evolution rate highly relies on the concentration of the Pt co‐catalyst (Figure S10), compensating the loss of the Pt co‐catalyst for the deactivated photocatalysts should be able to recover the performance.[^11^, ^12^] For the deactivated Pt/ICN, we confirm that the H_2_ evolution rate increases after adding a calculated amount of H_2_PtCl_6_ (equal to 1.0 w t% of Pt loaded on ICN) into the fresh solution for the second‐cycle test (Figure 3e). Therefore, the decrease of active sites (Pt co‐catalyst) for H_2_ evolution is the major reason for the deactivation of Pt/ICN.

The finding of the photocatalytic instability of the carbon nitride based photocatalyst is surprising because many research works have reported stable operations of Pt/carbon nitride based photocatalysts for at least 24 hours.[^21^, ^22^] We supposed that this “discrepancy” between our observation and the previous reports could be attributed to the different reaction conditions in these photocatalytic measurements.^[23]^ In this regard, more systematic photocatalytic tests were conducted under different reaction conditions to find the reason for the detachment of the Pt co‐catalyst from the surface of ICN.

**Figure 4:**
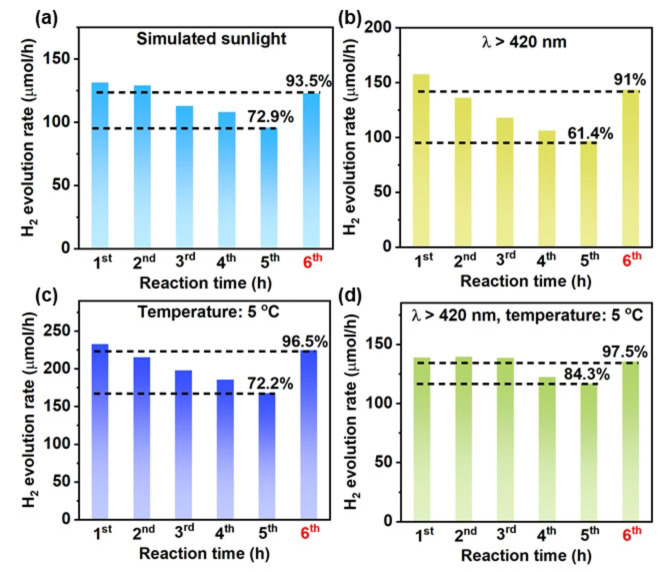
Photocatalytic H_2_ evolution activity of Pt/ICN under different reaction conditions. The reaction condition is the same as the ‘accelerated test’ except for the noted one. (a) Simulated sunlight; (b) Light wavelength λ>420 nm; (c) Reaction temperature 5 °C; (d) Light wavelength λ>420 nm, reaction temperature 5 °C. For the H_2_ evolution rate at the 6^th^ hour, the catalyst was collected after the 5^th^ hour and re‐dispersed in a fresh solution for the photocatalytic H_2_ evolution reaction under the same test condition.

Interestingly, the H_2_ evolution rates remain over 90 % of the initial value after being re‐dispersed in a fresh solution when the photocatalytic measurements were performed under simulated sunlight, visible light (>420 nm) and low temperature, respectively (Figure 4a–c). In particular, 84.3 % of the initial H_2_ evolution activity was maintained after five‐hour testing under the irradiation of visible light (>420 nm) at 5 °C and the H_2_ evolution rate recovered to 97.5 % of the initial value after being re‐dispersed in a fresh solution (Figure 4d). The Pt content of tested Pt/ICN is 2.89 %, which is much higher than that of 1.03 % under the irradiation of a high‐intensity Xe lamp at 20 °C (Table S1). These results demonstrate the photostability of Pt/ICN under mild reaction conditions, which are consistent with the previous reports.[^5^, ^6^, ^24^, ^25^] It is worth noting that the initial H_2_ evolution rates under those conditions are much lower than that of the ‘accelerated test’, due to thermodynamic and kinetic impacts induced by different conditions.^[26]^ Therefore, it is mostly possible that the high reaction rate induced by the high‐intensity light is responsible for the deactivation of Pt/ICN in this work. In addition, the impact of the sacrificial electron donor on the deactivation processes was investigated by replacing TEOA with methanol. A slower decay of the H_2_ evolution activity together with a much lower H_2_ evolution rate was observed when replacing TEOA with methanol (Figure S11).^[18]^ In addition to the low H_2_ evolution rate, the slower decay with methanol may be attributed to the different physiochemical properties between methanol and TEOA.

**Figure 5:**
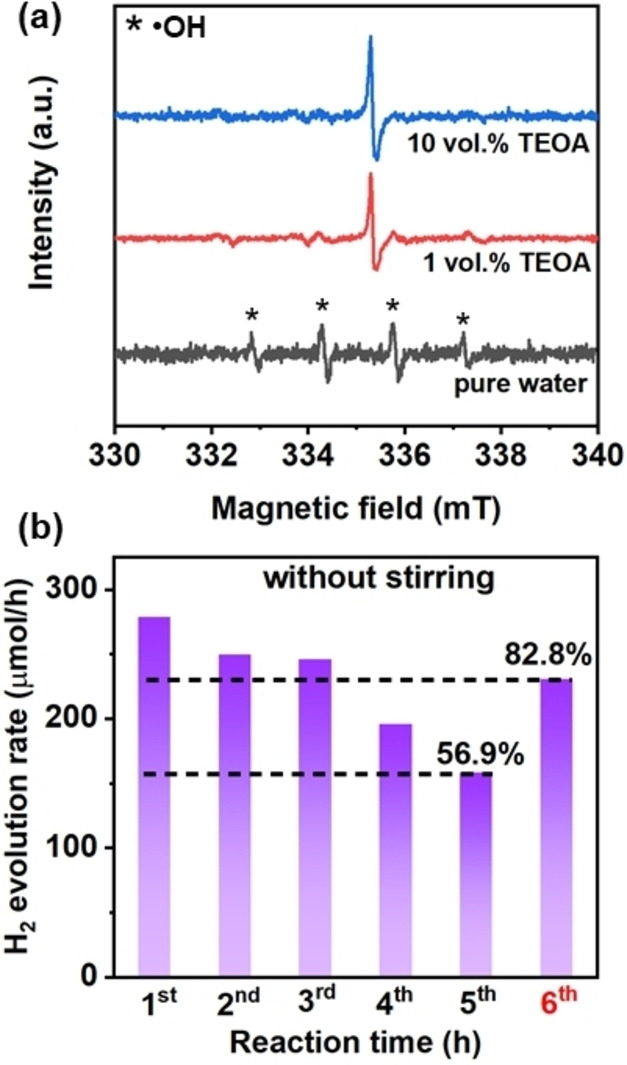
(a) In‐situ EPR spectra of Pt/ICN in different solutions under high‐intensity light irradiation. The signal located near 335.5 mT may be related to the photo‐oxidation of TEOA. (b) Photocatalytic H_2_ evolution activity on Pt/ICN without mechanical stirring. For the H_2_ evolution rate at the 6^th^ hour, the catalyst was collected after the 5^th^ hour and re‐dispersed in a fresh solution for the photocatalytic H_2_ evolution reaction under the same test condition.

For the photocatalytic H_2_ evolution process on Pt/ICN, the photo‐generated electrons reduce the proton (H^+^) to produce H_2_, while the holes are generally consumed by TEOA.^[27]^ Compared to standard sunlight, high‐intensity light markedly accelerates the photocatalytic H_2_ evolution rate, as more photons can be converted to charge carriers in Pt/ICN for photocatalytic reactions.^[28]^ This enhanced H_2_ production may lead to a higher probability that the H_2_ gas bubbles adsorb on active sites of the photocatalyst, which in turn reduces the direct contact between the photocatalyst and the reactants.^[29]^ As a result, the photo‐charged electrons and holes may accumulate within the photocatalyst and further induce self‐oxidation/reduction of the photocatalyst itself, which is so‐called photo‐corrosion.^[26]^ In general, the hydroxyl radical (⋅OH) is responsible for the decomposition of carbon nitride photocatalysts, as ⋅OH can transform the heptazine unit of the ICN framework to soluble nitrates in the aqueous environment.[^8^, ^9^, ^10^] If the photo‐charged holes accumulate at the interface of ICN and Pt, ⋅OH can be generated by the reaction between photo‐generated holes and the adsorbed hydroxyl groups on the surface of ICN.[^8^, ^9^, ^10^, ^30^, ^31^, ^32^] As shown in Figure 5a, hydroxyl radicals (⋅OH) are detected on Pt/ICN in pure water, while no ⋅OH radicals are detected in the 10 % TEOA aqueous solution, suggesting efficient consumption of photo‐generated holes can be achieved given sufficient access to TEOA. Therefore, these results indicate that ⋅OH can be generated at the interface of ICN and Pt where the photo‐charged holes accumulate and meanwhile, there is limited or no access to sacrificial electron donors.[^33^, ^34^] Even though ⋅OH can lead to the decomposition of carbon nitride frameworks, negligible structural changes were observed in this work, probably due to the relatively mild photo‐corrosion that only weakens the connection between the ICN and Pt nanoparticles, resulting in the detachment of Pt nanoparticles from the ICN.

Mechanical stirring may also have detrimental impacts on the surface of photocatalysts during the testing, and thus a comparison study was carried out to evaluate this parameter.[^11^, ^12^, ^35^] As a benchmark system, the original Pt/ICN photocatalyst was kept stirring for 5 hours in the dark and then collected for elemental analysis. The Pt content of this Pt/ICN is 2.93 %, which is comparable to the value in the initial Pt/ICN. It seems that the collision with the magnetic stirrer itself cannot significantly remove Pt co‐catalysts from the surface of ICN. Furthermore, a photocatalytic H_2_ evolution test on Pt/ICN without mechanical stirring was conducted. The Pt content of the Pt/ICN is 2.53 % after a five‐hour reaction and 82.8 % of the initial H_2_ evolution rate remains after being re‐dispersed in a fresh solution (Figure 5b), both of which are much higher than that in the presence of stirring (Figure 2). These results suggest that the mechanical stirring promotes the detachment of Pt nanoparticles on ICN during photocatalytic H_2_ evolution under the irradiation of high‐intensity light.[^11^, ^12^]

**Figure 6:**
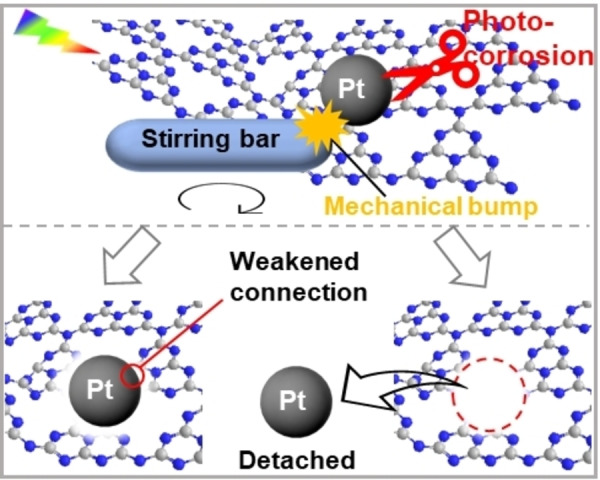
Scheme of the proposed deactivation mechanism of the Pt/ICN photocatalyst during the H_2_ evolution reaction.

Based on the aforementioned discussion, a possible mechanism is proposed to explain the deactivation of the Pt/ICN photocatalyst during the photocatalytic H_2_ evolution reaction (Figure 6). Upon intensive light irradiation, photo‐generated electrons are transferred to Pt nanoparticles for the H_2_ evolution reaction, and the high H_2_ productivity may subsequently lead to the adsorption of H_2_ bubbles on the surface of Pt/ICN, thus hindering the access of reactants (particularly the sacrificial electron donor) to the Pt/ICN surface active sites. As a result, the excess photo‐generated holes induce photo‐degradation of the carbon nitride frameworks because of limited access to sacrificial electron donors.[^8^, ^9^, ^10^] The decomposition of ICN together with the mechanical collisions from the magnetic stirring bar, weakens the interfacial bonding between the Pt nanoparticle and the ICN framework and eventually leads to the detachment of Pt nanoparticles from the surface of ICN. Accordingly, some recommendations are proposed to suppress the deactivation. 1) Further modification of the carbon nitride frameworks may be helpful to suppress the photo‐corrosion and improve the structural stability.[^36^, ^37^] 2) Surface engineering of the photocatalyst can be applied to promote the desorption of H_2_.^[38]^ 3) The photocatalyst sheet can be prepared to avoid the detrimental impact of mechanical stirring on the photocatalyst.^[20]^ 4) Flow‐type photocatalytic system can be a better design to promote the removal of products (*i. e*. H_2_ bubbles) from the surface of photocatalysts.

## Conclusions

This work unveils the origins of the deactivation of carbon‐nitride‐based photocatalysts for solar H_2_ evolution under ‘accelerated’ testing conditions. The deactivation of carbon nitride photocatalysts can be mainly attributed to the detachment of the Pt co‐catalyst from the surface of carbon nitride, due to a synergistic effect of photo‐corrosion and mechanical stirring during the operation. These findings not only inform the key influencing parameters that limit the long‐term operational stability of carbon nitride photocatalysts but also provide insights into understanding the photostability of many other polymeric photocatalysts, for example, metal‐organic frameworks and covalent‐organic frameworks. Based on the deactivation mechanism, possible strategies are proposed to improve the stability of photocatalysts for liquid‐phase heterogeneous photocatalysis. Furthermore, we also demonstrate that high‐intensity light illumination can be used as an effective strategy for an ‘accelerated test’ of the operational stability of photocatalysts. Such ‘accelerated test’ can facilitate the rapid identification of deactivation problems, thus speeding up the development of stable photocatalysts. This case study provides very useful guidance for the future design of photocatalysts and photocatalytic systems towards more efficient and stable solar‐to‐hydrogen conversion.

## Experimental Section

**Chemicals**. Potassium chloride (KCl, Sigma‐Aldrich, P3911), Lithium chloride (LiCl, Sigma‐Aldrich, 203637), Melamine (Sigma‐Aldrich, M2659), Urea (Sigma‐Aldrich, U5128), Titanium dioxide (TiO_2_, anatase, Sigma‐Aldrich, 637254), Chloroplatinic acid hexahydrate (H_2_PtCl_6_ ⋅ 6H_2_O, Sigma‐Aldrich, 206083), Triethanolamine (TEOA, Sigma‐Aldrich, 90279), Methanol (Merck, 106009), 5,5‐Dimethyl‐1‐pyrroline *N*‐oxide (DMPO, Sigma‐Aldrich, 92688, for ESR‐spectroscopy), Dimethyl sulfoxide (Sigma‐Aldrich, 472301), Deionized (DI) water. All chemicals were used without further purification.

**Material synthesis**. Three typical types of carbon nitride materials were fabricated using different precursors and methods. 1) Covalent carbon nitride materials were synthesized through direct thermal polymerization (550 °C, 4 h) of urea and melamine, which were named UCN and MCN, respectively.^[3]^ 2) The ionic carbon nitride (ICN) was prepared using a modified ionothermal synthesis.[^5^, ^6^] In detail, 10 g of urea was calcined at 500 °C for 1 h in air. Next, the pre‐treated urea precursor was mixed with 5.5 g of KCl and 4.5 g of LiCl, followed by further calcination at 550 °C for 2 h in an N_2_ atmosphere. After cooling down naturally, the product was washed 5 times using deionized water to remove alkali salts and collected via a vacuum filter. The obtained samples were dried at 60 °C for 12 h before further applications.

**Characterisation**. The chemical structure of materials was analysed using X‐ray diffraction (XRD, Cu Kα X‐ray source, Rigaku), the Fourier‐transform infrared spectroscopy (FTIR, ThermoFisher Scientific), and the X‐ray photoelectron spectroscopy (XPS, Al Kα X‐ray source, AXIS Supra+, Kratos Analytical). The C 1s peak with a binding energy of 284.8 eV was used as a standard to calibrate the XPS spectra. The morphology of photocatalysts was characterized by high‐resolution transmission electron microscopy (HRTEM, Hitachi HF5000) and scanning electron microscopy (SEM, JEOL JSM‐7800F). The loading amount of the Pt co‐catalyst on carbon nitride photocatalysts was quantified via the inductively coupled plasma optical emission spectroscopy (ICP‐OES). The light‐absorption property of photocatalysts was characterized by the ultraviolet (UV)‐vis spectrophotometer (V‐650, Jasco). The in‐situ electron paramagnetic resonance (EPR) measurement was conducted on a Bruker Elexsys E500 CW EPR spectrometer.^[39]^ Typically, 10 mg of 5,5‐Dimethyl‐1‐pyrroline N‐oxide (DMPO) was dissolved in 1 mL of dimethyl sulfoxide (DMSO) as solution A and 1.0 mg/mL of the photocatalyst dispersed in solution (water with/without triethanolamine, TEOA) was prepared as suspension B. Next, 5 μL of solution A and 50 μL of suspension B were mixed, followed by injecting 15 μL of the mixture into a glass capillary tube for the EPR measurement under the irradiation of the same Xe lamp used for photocatalytic measurement.

**Photocatalytic measurement**. The photocatalytic H_2_ evolution reaction was performed in an air‐tight reactor with a quartz window on the top side. A high‐intensity Xe lamp (PLS‐SXE300D, PerfectLight) was used as the light source, of which the intensity and wavelength range of the light were adjusted by using different optical filters. The produced H_2_ gas was quantified by a gas chromatography (GC‐2014, Shimadzu) system connected to the reactor, which was equipped with a molesieve 5 A packed column (Restek) and a thermal conductivity detector (TCD). The photocatalytic powder was dispersed in reactant solution via ultrasonication and the obtained suspension was transferred into the reactor, followed by the purge of Argon gas for 1 h to remove air in the reactor. Platinum (Pt) as the co‐catalyst was loaded on the photocatalyst via an in‐situ photo‐deposition method, of which the chloroplatinic acid (H_2_PtCl_6_) was used as the precursor.[^5^, ^6^] The temperature of the reactor was kept at 20 °C by a thermostatic system to minimize the light‐induced temperature fluctuation. In addition, a magnetic stirring with a spin rate of 200 rpm/min was operated to keep the suspension of photocatalyst particles during the reaction if there was no specific notification. After the photocatalytic reaction, the photocatalyst was washed using water, collected via a vacuum filter, and dried in a vacuum oven at room temperature for further characterization and cycling tests.

Titanium dioxide (TiO_2_) photocatalyst: 50 mg of TiO_2_ powder was dispersed in 50 mL of 10 vol % methanol aqueous solution, together with a calculated amount of H_2_PtCl_6_ (equal to 1.0 wt . % Pt on TiO_2_).

Carbon nitride photocatalysts: in a typical procedure, 20 mg of carbon nitride materials were dispersed in 50 mL of an aqueous solution containing 10 vol % of TEOA or methanol. together with a calculated amount of H_2_PtCl_6_ (equal to 3.0 wt . % Pt on carbon nitride).

## Conflict of Interests

The authors declare no conflict of interest.

1

## Supporting information

As a service to our authors and readers, this journal provides supporting information supplied by the authors. Such materials are peer reviewed and may be re‐organized for online delivery, but are not copy‐edited or typeset. Technical support issues arising from supporting information (other than missing files) should be addressed to the authors.

Supporting Information

## Data Availability

The data that support the findings of this study are available from the corresponding author upon reasonable request.
