# Supplementary material for: ‘Accelerated’ Deactivation of Carbon Nitride Photocatalyst for Solar Hydrogen Evolution
Source: ChemSusChem. 2024 Aug 7;17(23):e202400937. doi: 10.1002/cssc.202400937 (PMC11632563; doi:10.1002/cssc.202400937)
Supplement: Supplementary file 1 — Supporting Information [file CSSC-17-e202400937-s001.pdf]

ChemSusChem

Supporting Information

‘Accelerated’ Deactivation of Carbon Nitride Photocatalyst for Solar Hydrogen Evolution

Mu Xiao,* Miaoqiang Lyu, Zitong Wang, and Lianzhou Wang*

Supporting Information for

'Accelerated' Deactivation of Carbon Nitride Photocatalyst for Solar Hydrogen Evolution

Mu Xiao, Miaoqiang Lyu, Zitong Wang, Lianzhou Wang

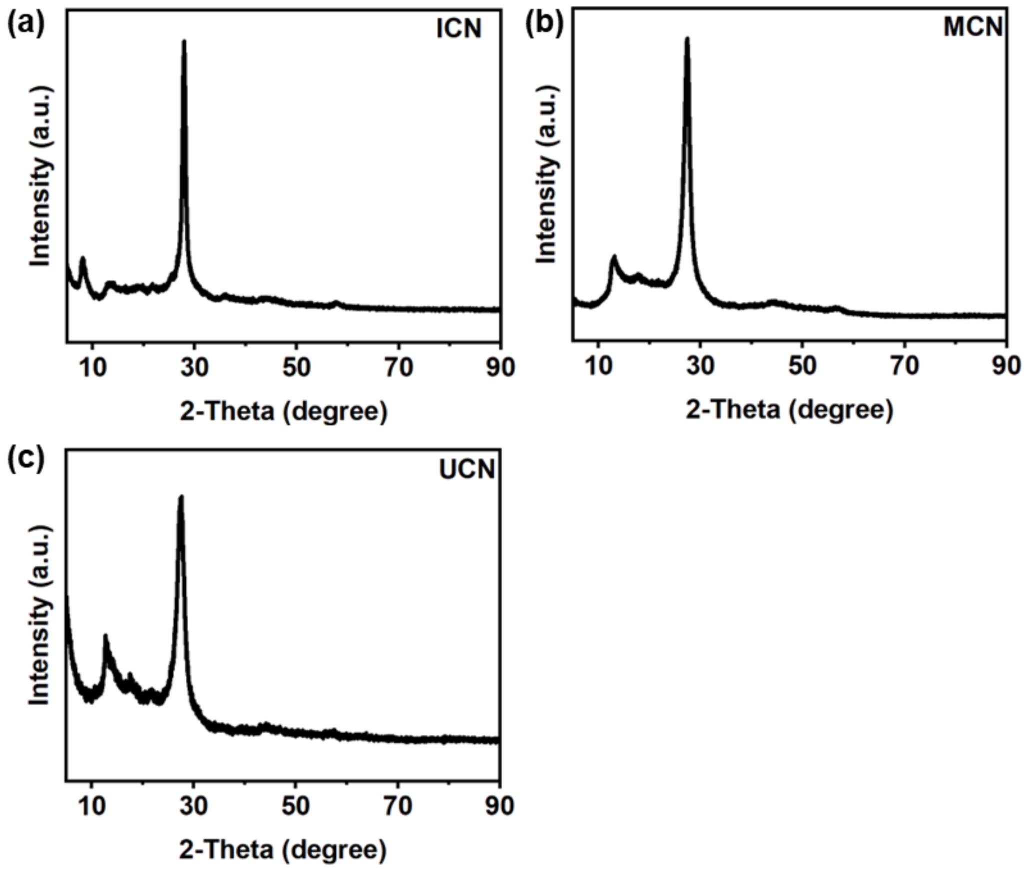

Figure S1. XRD patterns of (a)ICN, (b) MCN, and (c) UCN.

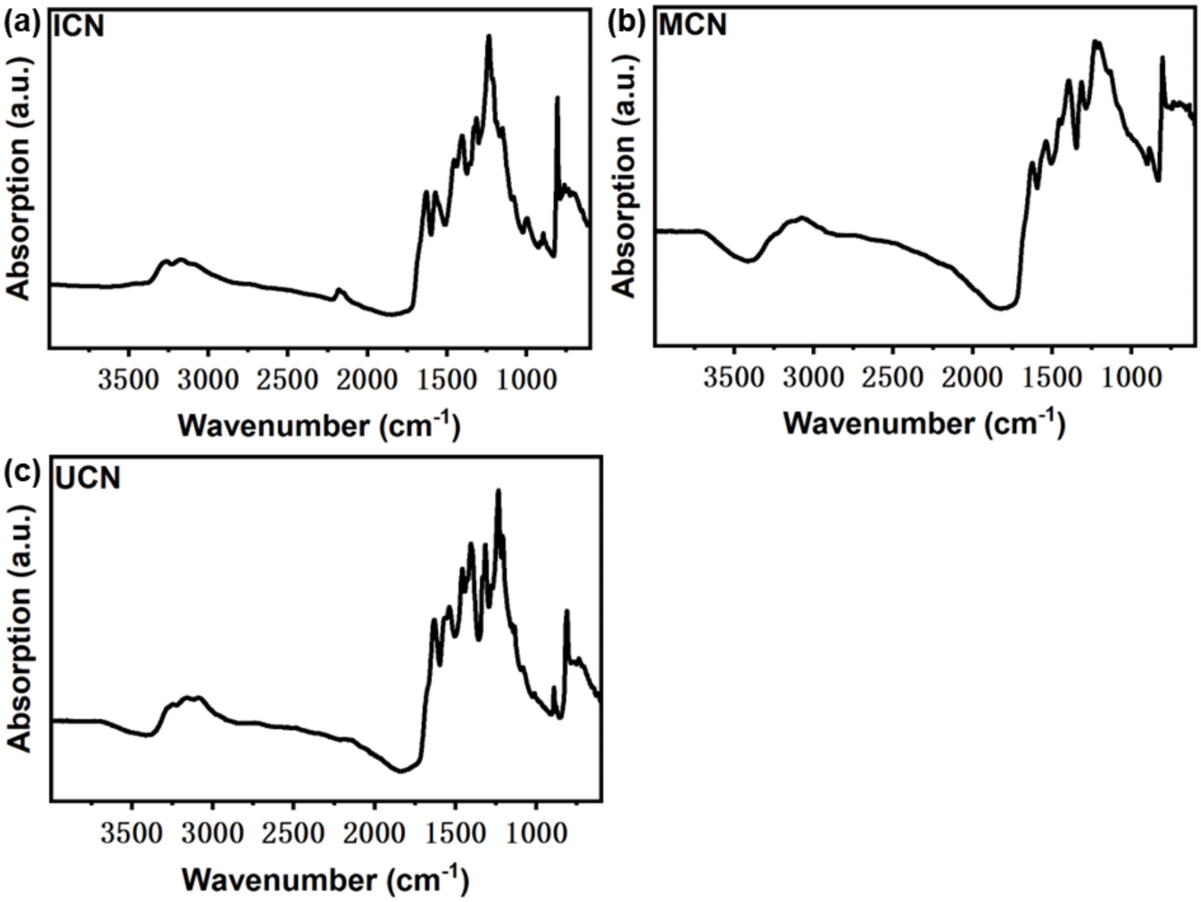

Figure S2. FTIR spectra of (a)ICN, (b) MCN, and (c) UCN.

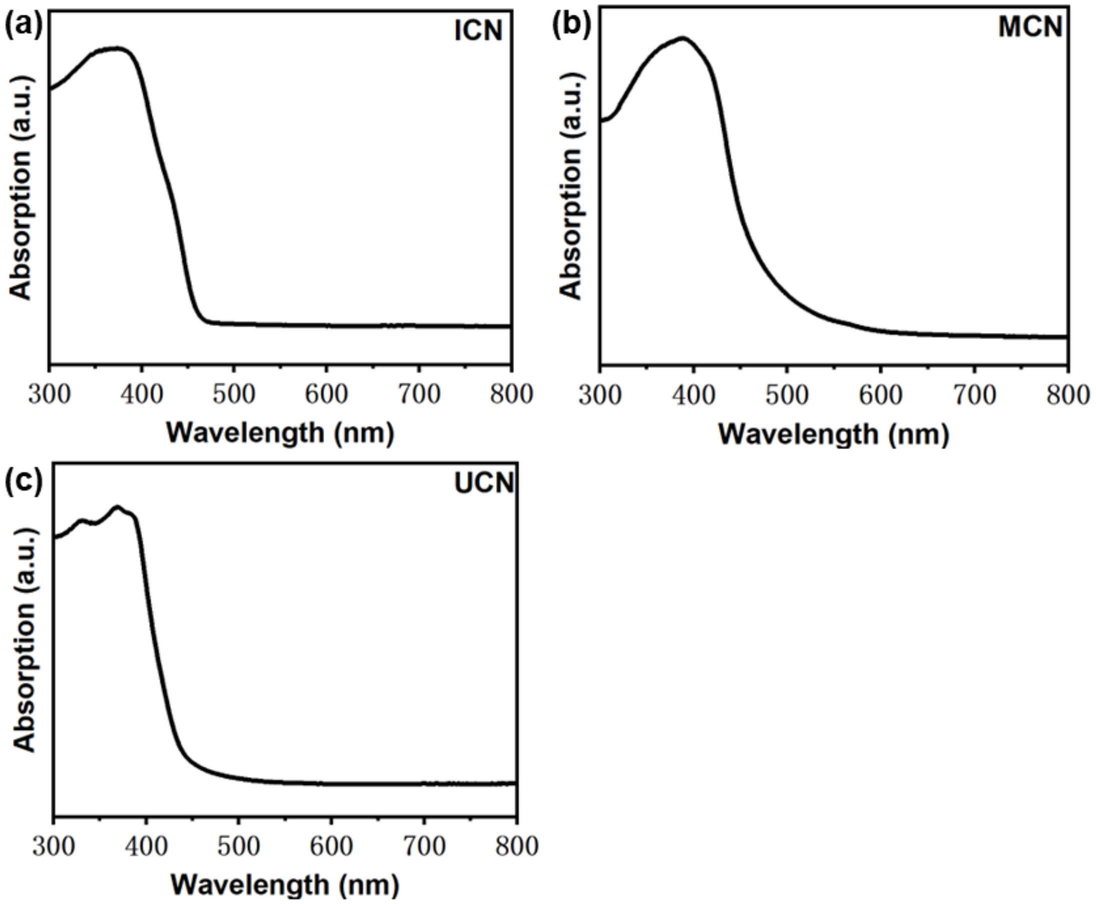

Figure S3. UV-vis absorption spectra of (a)ICN, (b) MCN, and (c) UCN.

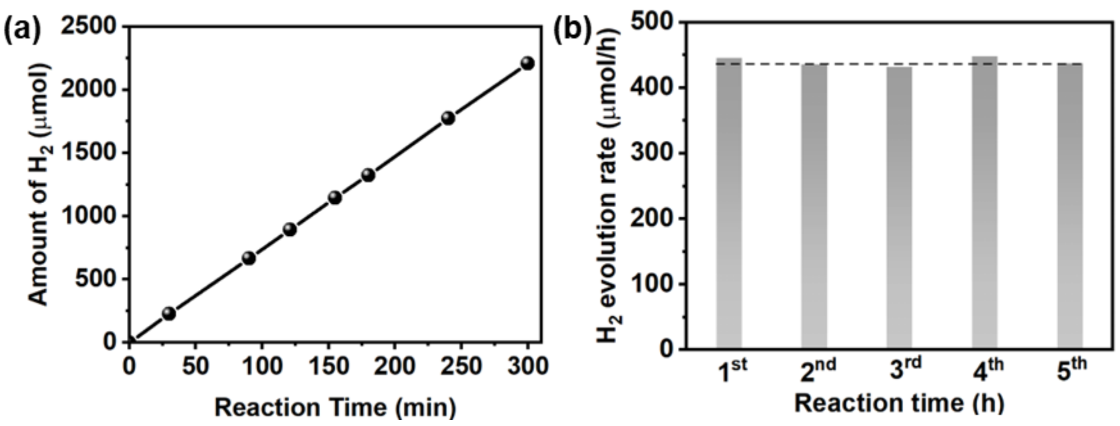

Figure S4. (a) Time-course photocatalytic H_2 evolution activity of Pt/TiO_2 and (b) the corresponding H_2 evolution rate.

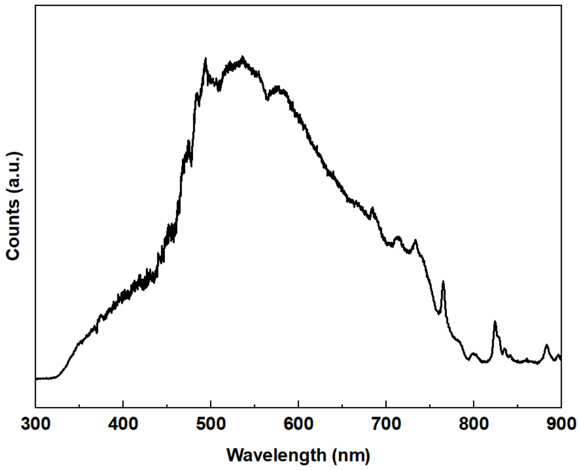

Figure S5. The light emission spectrum of the Xenon lamp (PLS-SXE300D, PerfectLight) used for the photocatalytic measurement. The working current was set as 12.5 A for all photocatalytic tests. The light intensity of the lamp was tested to be ~610 mW/cm^2, which is five times higher than the intensity (~100 mW/cm^2) of standard sunlight (AM1.5G).

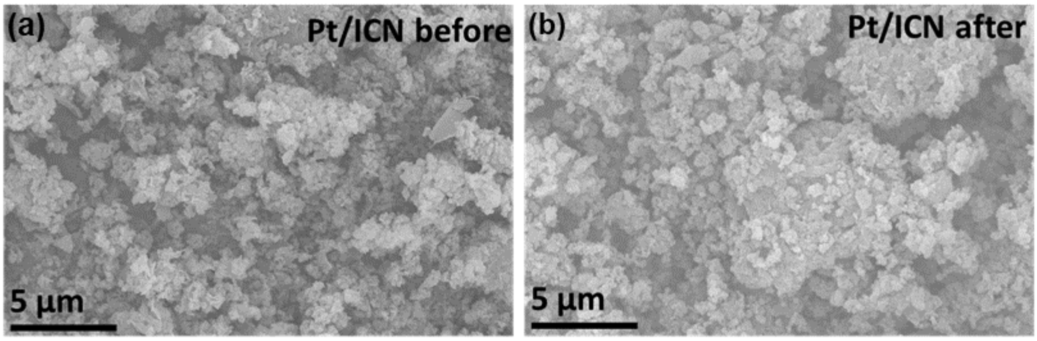

Figure S6. SEM images of Pt/ICN photocatalyst (a) before and (b) after the 5-h photocatalytic H_2 evolution reaction.

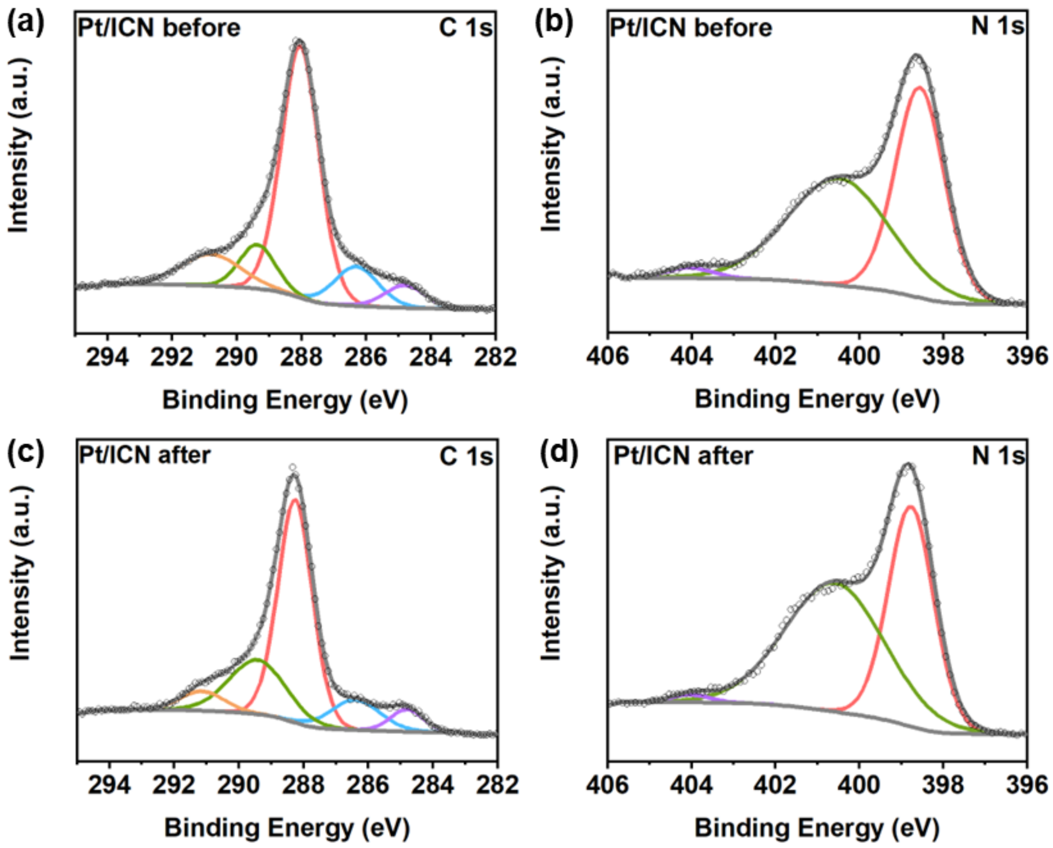

Figure S7. XPS spectra of Pt/ICN before (a, b) and after (c, d) the 5h-photocatalytic H_2 test.

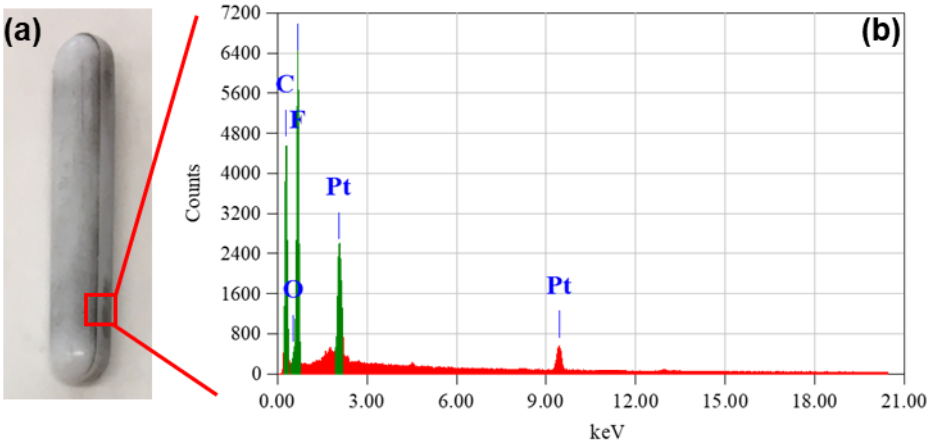

Figure S8. (a) Stirring bar and (b) the corresponding EDS analysis.

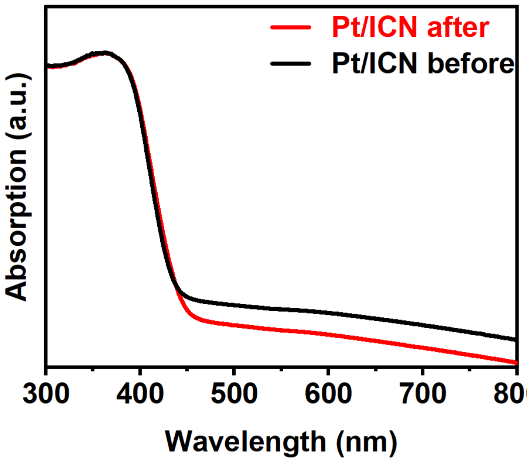

Figure S9. UV-vis absorption spectra of Pt/ICN before and after the 5h-photocatalytic H_2 evolution reaction, respectively.

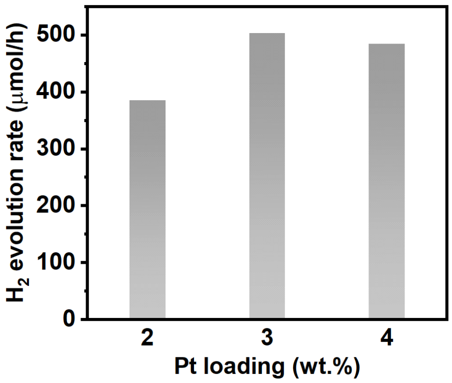

Figure S10. Photocatalytic H_2 evolution rate of Pt/ICN with different Pt contents.

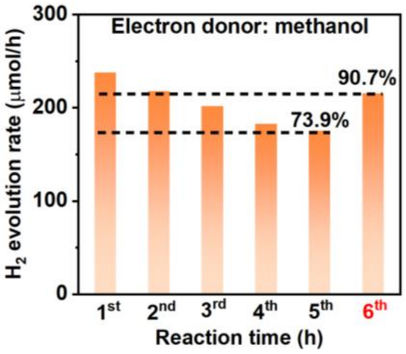

Figure S11. Photocatalytic H_2 evolution activity of Pt/ICN in 10 vol.% methanol aqueous solution.

Table S1. Pt content of various Pt/ICN samples analysed by ICP-OES.

Sample	Pt content (wt.%)
Pt/ICN original (after photo-deposition)	3.04
Pt/ICN after 5-h reaction	1.03
Pt/ICN after 5-h reaction (5 °C, > 420 nm)	2.89
Pt/ICN after 5-h reaction (solar simulator)	2.72
Pt/ICN after 5-h reaction (unstirring)	2.53
Pt/ICN after stirring under dark for 5 h	2.93
